# Immune System Modulation by the Adjuvants Poly (I:C) and Montanide ISA 720

**DOI:** 10.3389/fimmu.2022.910022

**Published:** 2022-06-29

**Authors:** Rodolfo F. Marques, Filipe Menegatti de Melo, Janaina Tenório Novais, Irene S. Soares, Daniel Youssef Bargieri, Alba Marina Gimenez

**Affiliations:** ^1^ Department of Parasitology, Institute of Biomedical Sciences, University of São Paulo, São Paulo, Brazil; ^2^ Department of Clinical and Toxicological Analyses, School of Pharmaceutical Sciences, University of São Paulo, São Paulo, Brazil

**Keywords:** montanide, poly (I:C), Plasmodium vivax, vaccine, immune response

## Abstract

Adjuvants are essential for vaccine development, especially subunit-based vaccines such as those containing recombinant proteins. Increasing the knowledge of the immune response mechanisms generated by adjuvants should facilitate the formulation of vaccines in the future. The present work describes the immune phenotypes induced by Poly (I:C) and Montanide ISA 720 in the context of mice immunization with a recombinant protein based on the *Plasmodium vivax *circumsporozoite protein (PvCSP) sequence. Mice immunized with the recombinant protein plus Montanide ISA 720 showed an overall more robust humoral response, inducing antibodies with greater avidity to the antigen. A general trend for mixed Th1/Th2 inflammatory cytokine profile was increased in Montanide-adjuvanted mice, while a balanced profile was observed in Poly (I:C)-adjuvanted mice. Montanide ISA 720 induced a gene signature in B lymphocytes characteristic of heme biosynthesis, suggesting increased differentiation to Plasma Cells. On the other hand, Poly (I:C) provoked more perturbations in T cell transcriptome. These results extend the understanding of the modulation of specific immune responses induced by different classes of adjuvants, and could support the optimization of subunit-based vaccines.

## Introduction

Malaria is a severe public health problem that affects countries with tropical and subtropical climates worldwide. In 2020, the World Health Organization (WHO) estimated that 240 million cases were registered in 87 endemic malaria countries, leading to about 627,000 deaths in the same year (1). Human malaria is caused by four human-specific *Plasmodium* species and some non-human primates-infecting species capable of producing zoonotic infections. Globally, while *P. falciparum* is responsible for the most deaths, *P. vivax* is the most geographically widespread (1).

Vaccination is undoubtedly among the public health interventions that have mainly contributed to preventing several life-threatening or disabling diseases caused by infectious agents (2). In the specific case of vaccines against protozoan parasites, such as *Plasmodium* spp, several factors hampered the development of effective formulations, like the complex life cycle of the parasites, antigenic variability, and poor immunogenicity of potentially protective antigens (3). In this sense, alternative adjuvants could be the key to obtaining effective vaccine formulations (4).

During vaccine development, it is not uncommon for clinical trial results to lead to the replacement of adjuvants by more efficient ones. A good example is the RTS,S vaccine, the first WHO-approved *P. falciparum* malaria vaccine for human use currently being implemented in African countries (1). This formulation is based on a virus-like particle that displays *P. falciparum* Circumsporozoite protein (CSP) sequences on the hepatitis B virus surface antigen (HBsAg) carrier. During its development, some adjuvants were tested to generate better protective responses. The first adjuvant tried was AS04, a combination of alum with monophosphoryl lipid A (MPL). It was subsequently replaced by AS02A, a mixture of an oil-in-water emulsion plus MPL and the saponin QS-21 from *Quillaja saponaria* extract. Finally, after numerous tests, AS01E, composed of QS-21 and 3-odesacyl-4’-MPL, was chosen. Even though its effectiveness is suboptimal (30%) and short-lived (decay in 4 years), this formulation could attenuate the malaria burden (5).

We previously developed CSP-based vaccine formulations against *P. vivax* malaria. The basic chimeric protein, PvCSP-All epitopes, is a fusion of the PvCSP conserved region I (RI) with the three central repeat regions of different PvCSP alleles (VK210, VK247, and *P. vivax*-like), and the PvCSP C-terminal region (6). Several adjuvants have been tested to enhance their immunogenicity (7, 8). One of them, Poly (I:C), elicited high antibodies titers conferring partial protection to immunized mice (6, 8, 9). Another adjuvant tested in our formulations was Montanide ISA 720, which generated even higher IgG titers and promoted a greater decline in parasite burden during the challenge test when compared to Poly (I:C)- adjuvanted formulations (10).

Thus, the different patterns in the immunological response generated by the effect of the adjuvants could directly influence the protective effect of each formulation, mainly because they have different mechanisms of action (10). Poly (I:C) is a synthetic double-stranded RNA (dsRNA) designed to act like a natural ligand of TLR3 receptor, and known to elicit long-lived antibodies with strong Th1 responses against *P. falciparum* antigen (11). On the other hand, Montanide ISA 720 is an oil-based emulsion dispersion that activates innate inflammatory responses and recruits antigen-presenting cells (APCs), enhancing the persistency of the antigen at the injection site, which favors the antigen delivery to immune cells but could also cause high reactogenicity (12, 13).

Increasing knowledge and research on understanding the mechanisms of the immune response generated by each vaccine should facilitate the rationale for choosing the best adjuvant in a formulation. For these reasons, in this work, we aimed to better understand the differential immune response profile favored by Poly (I:C) and Montanide ISA 720 in mice immunized with formulations containing PvCSP-All epitopes as antigen. To this end, we analyzed IgG antibodies and cytokine profiles triggered by the formulations; and compared the transcriptome of the lymphocyte populations to understand the activated pathways and possible mechanisms of action of each adjuvant. We found that Montanide induced higher titers of antibodies against PvCSP and, more important, antibodies that have higher avidity to the target antigen. This fact may be a consequence of a gene signature of heme biosynthesis expressed by the B cells, which is associated with the development of Plasma Cells.

## Experimental Procedures

### Production of PvCSP

Clones of *P. pastoris* yeast previously selected to express the recombinant protein yPvCSP-All_CT_ (6) (hereafter PvCSP) were grown for 24 hours at 30°C with constant agitation (230 rpm) in 40-200 mL of buffered complex glycerol medium (BMGY). The cells were then harvested by centrifugation, resuspended in 40-200 mL of buffered complex methanol medium (BMMY), and cultured at 28°C with constant agitation (230 rpm) to enable the expression of the recombinant protein. Induction was maintained by the daily addition of 1% methanol throughout the 72-96 hours incubation period. The cells were harvested by centrifugation, and the supernatant was filtered out using 0.45μm membranes (Merck Millipore, MA, USA).

### Purification of Recombinant Proteins

The purification of the recombinant proteins was performed in a two-step procedure (affinity and ion-exchange chromatography). The supernatant containing the solubilized protein was subjected to affinity chromatography using a HisTrap™ FF nickel column coupled to the FPLC ÄKTA prime plus system (GE Healthcare USA Inc., Pittsburgh, PA). Elution occurred against an imidazole gradient (15-400 mM) in a phosphate-chloride buffer [20 mM NaH_2_PO_4_, 20 mM Na_2_HPO_4_, 0.5 M NaCl, pH=6.0].

Fractions containing the recombinant proteins, identified on 12% SDS-PAGE gels stained with Coomassie blue solution, were dialyzed on 10,000 MWCO SnakeSkin Pleated Dialysis Tubing membrane (Thermo Fisher Scientific USA Inc., Waltham, MA) against 20 mM Tris-HCl, (pH=8.0). After dialysis, the proteins were filtered (0.45 μm) and subjected to a purification step by ion-exchange chromatography using the HiTrap™ QFF Column (GE Healthcare USA Inc.), coupled to the ÄKTA system. Protein elution occurred in a linear gradient from 0 to 1M NaCl over 20mM Tris-HCl buffer. Fractions containing the chimeric recombinant proteins were dialyzed against phosphate-buffered saline (PBS) (8 mM NaH_2_PO_4_, 2.3 mM Na_2_HPO_4_, 130 mM NaCl, pH=7.4) overnight, with constant stirring at 4°C.

Protein concentration was determined by densitometry analysis using ImageQuant™ TL version 8.1 software (GE Healthcare USA Inc.) and compared to a calibration curve with defined concentrations of bovine serum albumin (BSA, Invitrogen, Life Technologies Corporation USA Inc.).

### Mice Immunization

Six- to eight-week-old female C57BL/6 mice (n=6 mice per group) were subcutaneously (s.c.) injected three times, two weeks apart, with one of the adjuvants [50 μg of Poly (I:C) HMW (Invivogen) or Montanide ISA 720 (Seppic), ratio 70/30 adjuvant/PBS] or immunized with recombinant protein plus adjuvant [10 µg of yPvCSP-All_CT_/PBS mixed with 50 μg of Poly (I:C) or Montanide ISA720 ratio 70/30 adjuvant/protein]. For each dose, a final volume of 100 µL was injected into the flank of each mouse. Mice were purchased from the School of Pharmaceutical Sciences/Chemistry Institute (University of Sao Paulo). All animal experiments were approved by the Animal Care and Use Committee of the University of São Paulo (CEUA/FCF 74.2016-P531).

### Serology Analysis

Fourteen days after each immunization, blood was obtained from the submandibular vein. Antibodies were detected by enzyme-linked immunosorbent assay (ELISA), as previously described (7). Sera were analyzed for the presence of antibodies against the homologous recombinant protein PvCSP (200ng/well). Following overnight incubation at room temperature (RT), the plates were washed with a solution of PBS 0.05% Tween-20 (PBS-T) and blocked with a blocking solution (PBS, 5% (w/v) skimmed milk) for 2 hours at 37°C. Serial dilutions starting with 1:200 murine polyclonal sera were added to the wells and incubated for 1 hour at RT. After a washing step with PBS-T, peroxidase-labeled goat anti-mouse IgG (Sigma, St. Louis, USA) was added to each well at a 1:3,000 dilution. Finally, revelation buffer (200 mM Na_2_HPO_4_, 200 mM citric acid, pH 4.7, 10% o-phenylenediamine dihydrochloride, 30% H_2_O_2_) was added for 15 min. The reaction was stopped with 4N H_2_SO_4,_ and the optical density (OD) was read at 490 nm using an ELISA plate reader (Thermo Scientific, Multiskan model 51119100). Titers were determined as the log of the last dilution with OD > 0.1. To detect IgG subclass responses, specific secondary antibodies to mouse IgG1, IgG2b, IgG2c, and IgG3 were used (Southern Technologies, Chattanooga, TN, USA). For the avidity assays, pooled sera were diluted to obtain an OD of ∼1.0. After the 2h incubation, the wells were treated for 30min in different concentrations of urea ranging from 6 to 0.5M in PBS. The plates were then washed for incubation with the secondary antibody and revelation as described above.

### Multiplex Assay for Cytokine Detection

Fourteen days after the last immunization, sera from immunized mice were processed following the manufacturer’s instructions. The test was performed using MILLIPLEX MAP Mouse Cytokine/Chemokine Magnetic Bead Panel - Premixed 32 Plex - Immunology Multiplex Assay, catalog number: MCYTMAG-70K-PX32. Multiplex assay was performed using the “Centro de Tecnologias Ômicas – CTO” facility, of the School of Pharmaceutical Sciences, University of São Paulo, SP, Brazil.

The following cytokines/chemokines were measured: Eotaxin/CCL11, G-CSF, GM-CSF, IFN-γ, IL-1α, IL-1β, IL-2, IL-3, IL-4, IL-5, IL-6, IL-7, IL-9, IL-10, IL-12 (p40), IL-12 (p70), IL-13, IL-15, IL-17, IP-10, KC, LIF, LIX, MCP-1, M-CSF, MIG, MIP-1α, MIP-1β, MIP-2, RANTES, TNF-α, VEGF.

Data acquisition was performed with the Luminex XPonent software for LX100/LX200 version 3.1.871.0 (Luminex Corporation, 2008 - Austin, Texas – USA). Five-parameter curve fitting (log scale) of Milliplex Analyst software version 3.5.5.0 was used for data analysis (copyright 2005, 2010 Vigene Tech Inc.).

### Analysis of Cellular Response Measured by Intracellular Cytokine Expression

To analyze the expression of intracellular cytokines, spleen cells harvested from mice fourteen days after the last immunization were used in intracellular cytokine staining (ICS) assays, as previously described (7). Briefly, 1×10^6^ splenocytes/well were plated in triplicates in U-shaped 96-well plates and stimulated with 10µg/mL of recombinant proteins representing the three variants of PvCSP (VK210, VK247 and *P. vivax*-like) (6). As a negative control, splenocytes were not pulsed. After incubation for one hour at 37°C and 5% CO_2_, Golgi Plug (Brefeldin A, BD Biosciences) was added to each well (0.5μg/well). Splenocytes were then incubated in the same conditions for 12 hours. Plates were centrifuged for 5min at 1,000×g and washed twice with PBS-FBS. Surface fluorescent antibody staining with mAbs αCD3-APC-Cy7 (clone 145-2C11), αCD4-PerCP-Cy5.5 (clone RM4-5), and αCD8-PE-Cy7 (clone 53-6.7), was performed. After three washes with PBS-FBS, cells were fixed and permeabilized for 15min using the Cytofix/Cytoperm kit (BD Biosciences). After three washes with PermWash buffer, the intracellular staining was performed on ice for 45min using the following mAbs: αIFNγ-APC (clone XMG1.2), αIL2-FITC (clone JES6-5H4), and αTNFα-PE (clone MP6-XT22). Cells were washed three times with PermWash buffer and resuspended in PBS-FBS. One million events were acquired in a FACS Canto II flow cytometer (BD biosciences). The percent of cytokine-producing cells was calculated after subtraction of the percent of cytokine-producing cells in the non-pulsed wells. CD4^+^ and CD8^+^ T cells were gated from CD3^+^ T lymphocyte population, as described elsewhere (14). Results were analyzed using the FlowJo program (Tree Star, Ashland, OR).

### Lymphocyte Isolation

Spleen cells from all experimental groups (n=6 per group) were harvested 14 days after the last dose and pooled before lymphocyte purification. The samples were subjected to three sequential sorting steps using Dynabeads ™ FlowComp ™ Mouse CD8, Dynabeads ™ FlowComp ™ Mouse CD4, and MagniSort ™ Mouse B cell Enrichment (ThermoFisher Scientific). The isolated cells were resuspended in RNAlater solution (ThermoFisher Scientific) and stored at -80°C before RNA purification.

### RNA Extraction, cDNA Library Preparation, and Sequencing

Total RNA purified from sorted lymphocyte populations was isolated and DNaseI treated using Qiagen RNeasy Mini Kit (Qiagen, Valencia, CA). RNA integrity was checked by Agilent Bioanalyzer 2100 and Agilent RNA 6000 Nano Chips (Agilent). The cDNA library was prepared by the company Quick Biology (Pasadena, CA, USA). Before cDNA synthesis, mRNA was prepared using the rRNA depletion technique. Libraries for RNA-seq were prepared according to KAPA Stranded RNA-Seq Kit with RiboErase (KAPA Biosystems, Wilmington, MA) system. Final library quality and quantity were analyzed by Agilent Bioanalyzer 2100 and Life Technologies Qubit3.0 Fluorometer, respectively. 150 bp Paired-end sequencing was performed on Illumina HiSeq 4000 (Illumnia Inc., San Diego, CA), with 24 million (24M) reads of sequencing depth.

### FastQ Files Alignment and Processing

Demultiplexed FastQ files were aligned against the UCSC Genome Browser Assembly GRCm38/mm10 *Mus musculus* genome with Hisat2. The list of splicing sites was also obtained from the UCSC genome browser, and it was provided to the algorithm during the alignment processing. Reads that aligned in more than one site, aligned to mitochondrial DNA, or were unaligned, were excluded using the Linux command line: sed ‘/chrM/d;/random/d;/chrUn/d;/XS:/d’. Resulting SAM files were converted to BAM using Samtools. These files were loaded to SeqMonk (Babraham Institute) and read quantification was performed against a probe that contained all the exons from mm10 mouse genome assembly. The probe was also generated in UCSC Genome Browser and genes were annotated according to NCBI RefSeq.

### Identification of Differentially Expressed Genes

Raw reads obtained from SeqMonk were used to calculate RPKM values from all aligned genes, and those that did not reach a minimum RPKM=3 in any of the samples were excluded from downstream analysis. Also, we used the raw reads to determine the Differentially Expressed genes using the R-Bioconductor DESeq2 package (15). Read 1 and Read 2 files from the paired-end sequencing of each sample were considered as replicates in DESeq2 calculations. Only genes that reached a p-adjusted < 0.05 were considered for downstream analysis. p values adjustment was obtained according to the Benjamini-Hochberg procedure of multiple hypothesis testing (16). Gene isoforms that showed the lower p-adjusted values were chosen and the others were omitted from downstream analysis. Volcano plots were plotted in R, while Venn diagrams were drawn in Microsoft PowerPoint according to the identification of common and exclusive genes from each sample performed in Microsoft Excel, using the “vlookup” function.

### Gene Enrichment Analyzes

Two strategies were used to perform Gene Enrichment Analysis. In the first one, the 4-fold induced genes by each adjuvant were submitted to the webtool Enrichr, which performs a Fisher exact test corrected by the standard deviation from the expected rank for each term in each gene-set library (17). We considered the retrieved results from four of the most cited libraries in literature, namely the Kyoto Encyclopedia of Genes and Genomes (KEGG) (18), WikiPathways (19), Reactome (20) and the Hallmark Gene Set Collection from The Molecular Signatures Database (MSigDB) (21). The retrieved terms from the four databases were ranked according to p-adjusted values and the most significant ones were plotted as -log (padj). Alternatively, analysis was performed using the Gene Set Enrichment Analysis (GSEA) Software from UCSD and Broad Institute (22), using normalized reads obtained from the above described DESeq2 analyses. GSEA software calculates the enrichment score (ES) that reflects the degree to which our list of genes is overrepresented at the top or the bottom of the evaluated libraries. The enrichment score corresponds to a weighted Kolmogorov–Smirnov-like statistic.

### Motif Analysis

Motif analysis was performed with the HOMER algorithm (23), using the “findMotifs.pl” command and the “mouse” promoter set included. Lists of the RefSeq numbers of the 3-fold induced genes were used as input. Although most analysis focused on 4-fold induced genes, we used the 3-fold for HOMER in an attempt to increase statistical power for some samples with few (less than 150) highly induced genes.

### Statistical Analysis

Other experiments were analyzed using Prism 8.0 (GraphPad, CA, USA). One-way analysis of variance (ANOVA) and Tukey’s honestly significant difference (HSD) test was used to compare the results from different groups. Differences were considered statistically significant when p<0.05.

## Results

### Montanide ISA 720 Induces Higher Titers of Antibodies With an Increased Binding Capacity to PvCSP

The immunogenicity of PvCSP was assessed after the immunization of mice with the recombinant protein in a formulation with Montanide ISA 720, or in conjugation with Poly (I:C). Each mouse received three doses of PvCSP, 14 days apart from each other, and sera were analyzed for the presence of antibodies against the homologous protein. It is worth mentioning that the recombinant PvCSP is a synthetic chimeric protein representing the three PvCSP alleles existing in the nature; whose specificity towards each allele was defined in previous works (6, 8).

**Figure 1:**
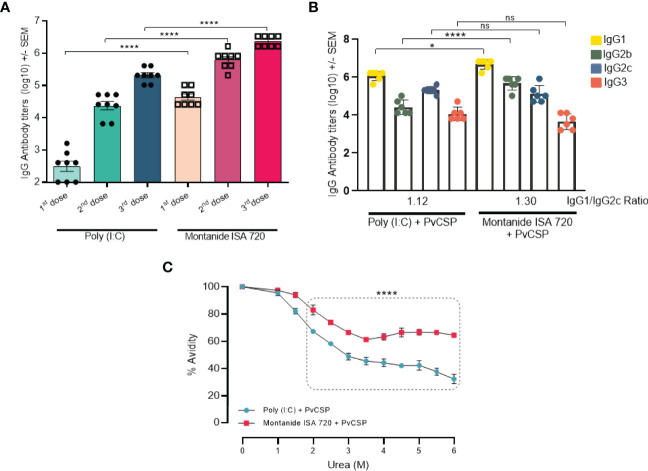
Montanide ISA 720 and Poly (I:C) elicit different antibody response profiles. C57BL/6 mice (n = 6) were vaccinated with three doses of PvCSP + Montanide ISA 720 or PvCSP + Poly (I:C). **(A)** PvCSP-specific IgG titers (Log) elicited 14 days after each immunizing dose were determined by ELISA. **(B)** Anti-PvCSP IgG1, IgG2b, IgG2c, and IgG3 subclasses were determined by ELISA 14 days after the last immunization. **(C)** Antibody affinity to PvCSP was measured after 30min of incubation in urea gradient solutions. Statistical significance was calculated on 6M urea point. Asterisks denote statistical differences; *p < 0.05; ****p < 0.0001; ns, not significant differences.

Montanide induced higher antigen specific IgG titer (10^5^) than Poly (I:C) (10^3^) even after only one immunization. Also, IgG titers after the third immunization were significantly higher for Montanide (p < 0.0001). Sera from mice immunized with Montanide + PvCSP showed titers of specific IgG reaching 10^7^, while mice immunized with Poly (I:C) + PvCSP barely crossed the barrier of 10^5^ (**Figure 1A**).

We then evaluated the quality of the antibody responses induced by the vaccine formulations. PvCSP-specific IgG subclasses and antibody avidity were analyzed in mice sera 14 days after the last immunization. Responses against the PvCSP protein resulted in Th1/Th2 balanced profile predicted for both adjuvants (p = 0.0845), with a slightly higher IgG1/IgG2c ratio (1.30) when Montanide was used as the adjuvant, when compared to Poly (I:C) plus PvCSP (IgG1/IgG2c ratio = 1.12). We also observed that the Montanide-adjuvanted group showed significantly higher titers of IgG1 (p = 0.0279) and IgG2b (p < 0.0001) than mice immunized with Poly (I:C) (**Figure 1B**). The presence of higher CSP-specific IgG2 titers (cytophilic antibodies) could be associated with protection against challenge, as previously reported in murine models (24).

Differences in the quality of the antibodies were more striking when we evaluated antibody avidity (**Figure 1C**). Sera from mice immunized with either Poly (I:C) + PvCSP or Montanide + PvCSP were incubated with increasing concentrations of urea for 30min. Statistically significant differences in avidity were already detected in samples incubated with 2.0M urea, with a greater drop in avidity detected in Poly (I:C) samples in comparison to Montanide. The maximum difference in avidity indexes was observed at 6.0M urea, where a drop from 100% to approximately 30% was observed in Poly (I:C) samples, while for Montanide the avidity index was reduced to only 60% (p<0.0001). Taken together, these data show that, although both vaccine formulations induce high antibodies titers, Montanide ISA 720 seems to be an overall better adjuvant since it not only induced higher titers of antibodies than Poly (I:C) but also antibodies with better biding capacity to the antigen.

### Poly (I:C)- and Montanide-Containing Vaccine Formulations Induce Different Cytokine Profiles in Sera of Immunized Mice

**Figure 2:**
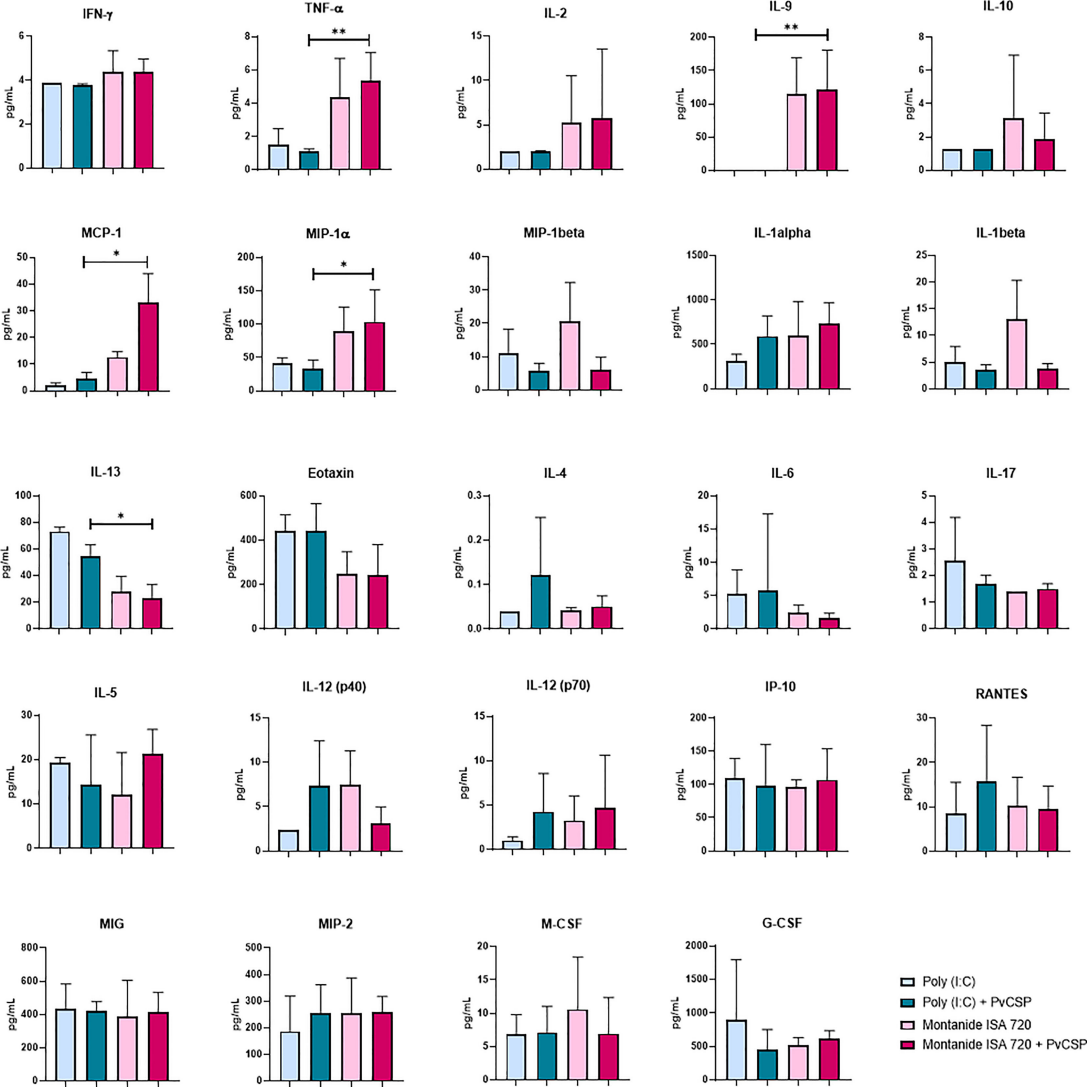
Montanide ISA 720 favors serum inflammatory cytokines, while Poly (I:C) elicits a balanced profile. The serum levels of cytokines were measured in mice sera obtained 14 days after the last immunization, by Luminex using the Milliplex MAP Mouse Cytokine/Chemokine Magnetic Bead Panel - Premixed 32 Plex. Sera samples were measured in triplicates; only the cytokines with the highest expression are depicted. Asterisks denote statistical differences; *p < 0.05; **p < 0.01.

To further investigate the immunological profiles induced by each adjuvant, we performed the quantification of 32 inflammatory and anti-inflammatory cytokines in mice sera obtained 14 days after the last immunization. Most of the evaluated cytokines were unaffected by vaccination, yet some interesting differences were detected (**Figure 2**). The Montanide-adjuvanted groups, independently of the presence of antigen, showed higher levels of tumor necrosis factor-alpha (TNF-α), monocyte chemoattractant protein 1 (MCP-1, also known as CCL-2), macrophage inflammatory protein-1 alpha (MIP-1α, also known as CCL-3), and interleukin (IL)-9, when compared to Poly (I:C). Interestingly, the presence of the antigen in the formulation provoked a significant decrease in IL-1β levels, compared to Montanide-only group (p = 0.0096).

On the other hand, Poly (I:C) induced higher levels of IL-13, a Th2 type cytokine, as well as a tendency to elicit other Th2 cytokines such as IL-6 and IL-4. No significant differences were observed for some important inflammatory cytokines that are understood to be essential for the protective effect of several vaccine formulations, like interferon-gamma (IFN-γ), IL-12, IL-17, or IL-2 (**Figure 2**).

In summary, our results show a general tendency of Th1/inflammatory cytokines to be increased in Montanide samples. However, the increment in IL-9 production suggest a mixed Th1/Th2 inflammatory profile, with characteristics of an allergic response (25). Similarly, a balanced profile is favored in Poly (I:C) adjuvanted samples, even though the elicited cytokines are different in both cases.

### Montanide Contributes to the Induction of CD4^+^ T Lymphocyte Clones That Produce IFN-γ and TNF-α

To determine whether immunization with the recombinant protein PvCSP elicited T cell-mediated immune responses, spleen cells were obtained from five immunized mice from each group. Splenocytes were re-stimulated *ex vivo* with the recombinant proteins representing the three PvCSP alleles. Twelve hours after culture, cells were intracellularly stained for IFN-γ, TNF-α, and IL-2. We detected a statistically significant increase in the frequency of CD4^+^ T cells that secreted IFN-γ (**Supplementary Figure 1A**) in samples from mice immunized with Montanide + PvCSP compared to samples from adjuvant-only injected mice. Also, the frequency of CD4^+^IFN-γ^+^ T cells was augmented in Montanide samples in comparison to Poly (I:C) samples, when lymphocytes were re-stimulated with PvCSP-210 or PvCSP-*P. vivax*-like alleles.

The frequency of CD4^+^TNF-α^+^ T cells from Montanide-adjuvanted groups was significantly higher than Poly (I:C)-adjuvated groups independently of the presence of antigen (**Supplementary Figure 1B**). No differences were observed among groups for CD4^+^IL-2^+^ T cells (**Supplementary Figure 1C**). Taken together, these ICS results show that Montanide-adjuvanted formulations induced CD4^+^IFN-γ^+^ T cells specific to PvCSP. Also, these results corroborated the findings depicted in **Figure 2**, in the sense that Montanide-immunized mice tend to show an inflammatory response with greater levels of TNF-α production, regardless of the presence of antigen.

The frequency of CD8^+^ T cells producing IFN-γ, TNF-α, or IL-2 was also analyzed. No significant differences were observed among the groups (data not shown).

### Montanide Induces a Gene Signature in B Lymphocytes Characteristic of Heme Biosynthesis, Suggesting Increased Differentiation to Plasma Cells

**Figure 3:**
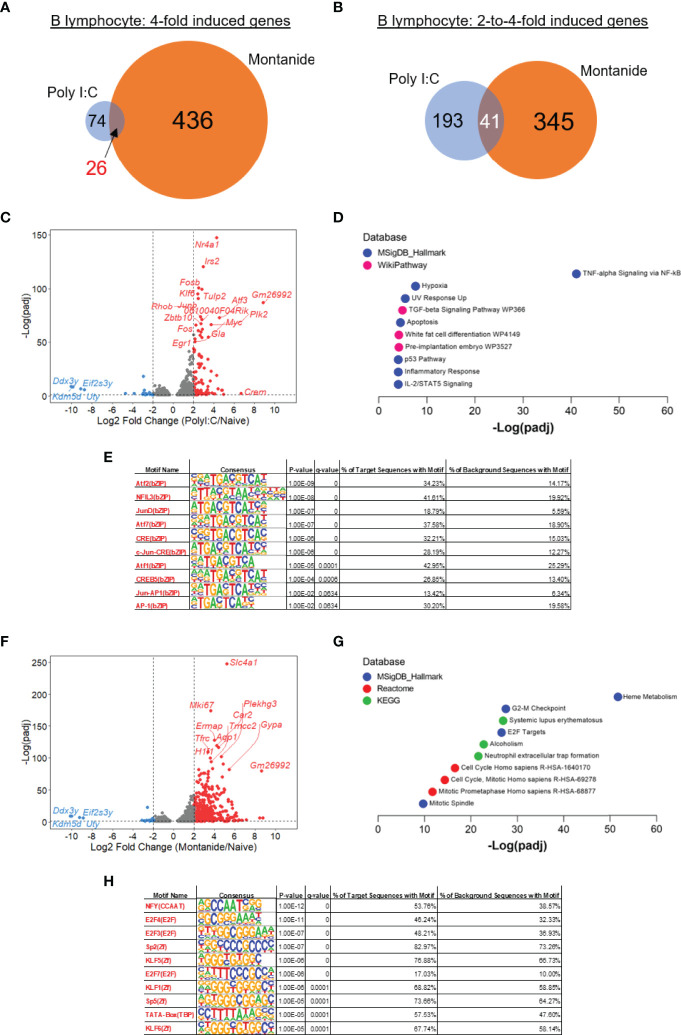
Transcriptome analysis of B cells show a strong “heme metabolism” gene signature in Montanide-adjuvanted mice. Differentially expressed genes (DEGs) were determined with the R-Bioconductor DESeq2 package, using the raw reads from the different samples as input. Genes that displayed a p-adjusted < 0.05 were considered for downstream analysis. **(A, B)**, Venn diagrams depicting DEGs detected only in Poly (I:C)-adjuvanted, only in Montanide-adjuvanted or in both groups of mice. **(C)**, volcano plot highlighting the Poly (I:C)-induced DEGs. **(D)**, gene enrichment analysis of the 4-fold-induced genes by Poly (I:C). **(E)**, HOMER motif analysis in the promoters of 3-fold induced genes by Poly (I:C), performed with the Enrichr webtool. **(F)**, volcano plot highlighting the Montanide-induced DEGs. **(G)**, gene enrichment analysis of the 4-fold-induced genes by Montanide ISA 720. **(H)**, HOMER motif analysis in the promoters of 3-fold induced genes by Montanide ISA 720, performed with the Enrichr webtool.

Transcriptome analysis from lymphocyte populations was initially focused on comparisons between samples derived from mice injected with adjuvant alone and naïve mice. Regarding B lymphocytes, Montanide seemed to induce greater changes in the transcriptome in comparison to Poly (I:C). The number of highly induced genes (at least 4-fold induced) in mice treated with Poly (I:C) was 74, while 436 genes were induced in Montanide-adjuvanted mice. Twenty-six genes were highly induced by both adjuvants (**Figure 3A**). Considering the pool of genes that were moderately induced (2-to-4-fold induction), 193 genes were induced in Poly (I:C)-adjuvanted mice, while 345 genes were induced in Montanide-injected ones. Forty-one genes were moderately induced by both adjuvants (**Figure 3B**). Volcano plots (**Figures 3C, F**) illustrate some of the genes that are induced in both cases.

We then performed Gene Enrichment Analysis, adopting two different approaches, and comparing the results. The first approach consisted in taking the 4-fold induced genes by each adjuvant and analyzing them with the webtool Enrichr. While the Poly (I:C) induced genes showed a great enrichment of genes related to “TNF-alpha Signaling *via* NF-κB” (**Figure 3D**), Montanide induced gene signatures related to Heme metabolism and Cell Cycle (**Figure 3G**), which indicate that B lymphocytes from Montanide-adjuvanted mice may have an intense proliferative activity and, also, may be largely differentiating in Plasma Cells (26). Among the most significantly induced genes by Montanide is *Tfrc* (**Figure 3F**), which encodes the transferrin receptor protein-1 (TFRC-1), essential for the importation of iron by cells and critical for lymphocyte development and proliferation (27).

Among the terms retrieved by the Gene Enrichment Analysis from the Poly (I:C)-induced genes (**Figure 3C**), the second most significant is “Hypoxia”, which is consistent with the fact that, in general, activated lymphocytes tend to skew their metabolism to a more glycolytic profile (28). Also, the “TGF-beta signaling pathway” emerged. TGF-β is a very important cytokine that fine tunes several B cell biological processes, like proliferation, cell death, antibody secretion and antibody class switching (29).

Besides the analyzes performed with Enrichr, we performed a second approach for Gene Enrichment using the GSEA software (22). Although the approach of performing stringent analyzes that consider only the highly expressed genes brings the opportunity to find unique molecular mechanisms that underlie the studied phenomena (30), we considered that it would also be interesting to perform an analysis that looks to the “big picture” of gene perturbations. This later approach assumes that even small modulations in genes (like 20% or 30% increases) may result in significant biological responses if the set of genes belongs to a particular pathway (22).

GSEA however largely confirmed the Enrichr analysis. Taking again the Hallmark Gene Set Collection from The Molecular Signatures Database, GSEA also retrieved the highest Enrichment Score (ES) for “TNF-alpha signaling *via* NF-κB” among the genes highly induced by Poly (I:C), while the highest ES in Montanide-adjuvanted sample was for the term “Heme Metabolism” (**Supplementary Figures 2A, B**). Of note, GSEA retrieved the term “mTORC1 signaling” for the Montanide sample (data not shown), and mTORC1 is essential for the endogenous synthesis of Heme by B cells that trigger the differentiation of Plasma Cells (31). “mTORC1” was not retrieved from the Enrichr analysis.

Finally, we performed motif analysis in the promoters of the highly induced genes by each adjuvant with the HOMER algorithm (23). For this analysis, we considered the 3-fold induced genes to increase the statistical power in the Poly (I:C) sample, since very few genes were induced by Poly (I:C) in B cells. The motifs enriched in Poly (I:C) highly induced genes were largely related to members of the AP-1 family of transcription factors (**Figure 3E**), which is consistent with the fact that some genes like *Fos, Fosb, Junb* and *Atf3* were among the most induced genes by Poly (I:C) and might indicate the involvement of MAPK signaling consequential to the activation of B cells (32). On the other hand, motif analysis in the promoters of Montanide-induced genes retrieved the enrichment of motifs mainly related to NFY transcription factor, which is a regulator of transcription that was already described to be involved in the enhanceosome of MHC II genes (33), and E2F transcription factors, which are involved in the activation of cell cycle genes (**Figure 3H**).

### Poly (I:C) Exerts More Perturbations in T Cell Transcriptome in Comparison to Montanide

**Figure 4:**
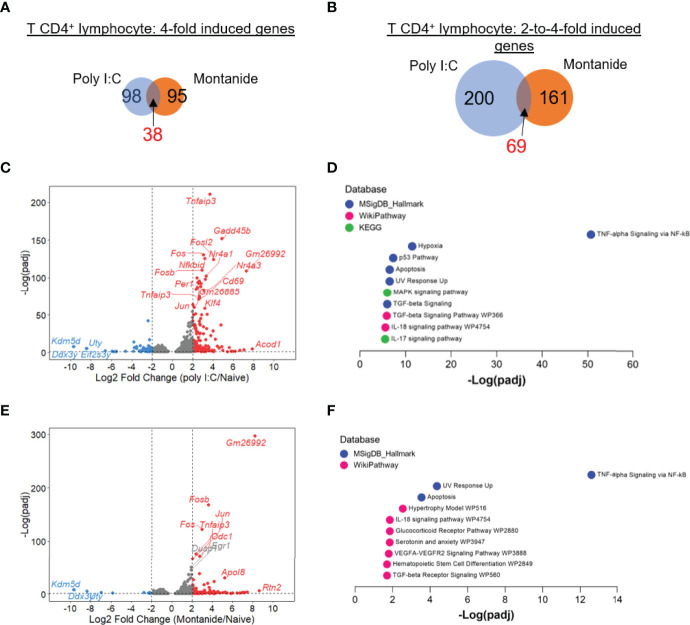
Poly (I:C) and Montanide ISA 720 induced almost the same number of DEGs in T CD4^+^ lymphocytes, although specific gene signatures were more significantly detected in Poly (I:C)-adjuvanted mice. Differentially expressed genes (DEGs) were determined with the R-Bioconductor DESeq2 package, using the raw reads from the different samples as input. Genes that displayed a p-adjusted < 0.05 were considered for downstream analysis. **(A, B)**, Venn diagrams depicting DEGs detected only in Poly (I:C)-adjuvanted, only in Montanide-adjuvanted or in both groups of mice. **(C)**, volcano plot highlighting the Poly (I:C)-induced DEGs. **(D)**, gene enrichment analysis of the 4-fold-induced genes by Poly (I:C), performed with the Enrichr webtool. **(E)**, volcano plot highlighting the Montanide-induced DEGs. **(F)**, gene enrichment analysis of the 4-fold-induced genes by Montanide ISA 720, performed with the Enrichr webtool.

We also analyzed the transcriptome of CD4^+^ and CD8^+^ T lymphocytes from mice treated with either adjuvant *versus* naïve mice. For both T cell populations, Poly (I:C) seemed to exert more prominent effects. Regarding CD4^+^ T cells, the number of 4-fold induced genes by either Poly (I:C) or Montanide were very similar (98 genes *vs* 95 genes, 38 genes induced by both adjuvants; **Figure 4A**). The number of 2-to-4-fold induced genes was also similar, with a slightly bigger number of genes in the Poly (I:C) sample (200 genes in Poly (I:C) sample *vs* 161 genes induced by Montanide, 69 genes were induced by both; **Figure 4B**). Volcano plots show the genes induced by Poly (I:C) (**Figure 4C**) and Montanide (**Figure 4E**).

Gene Enrichment Analysis of the highly induced genes (Enrichr analysis) retrieved the same top term for both adjuvants (“TNF-alpha signaling by NF-κB”), although the level of significance was much higher in Poly (I:C) sample (**Figures 4D, F**). Other terms that appeared in the enrichment analysis for Poly (I:C) sample included “Hypoxia”, “MAPK signaling”, “TGF-beta signaling”, “IL-17 signaling” and “IL-18 signaling” (**Figure 4D**). Some of these terms also appeared in Gene Enrichment analysis from the Montanide sample, but with a very lower level of significance (**Figure 4F**).

**Figure 5:**
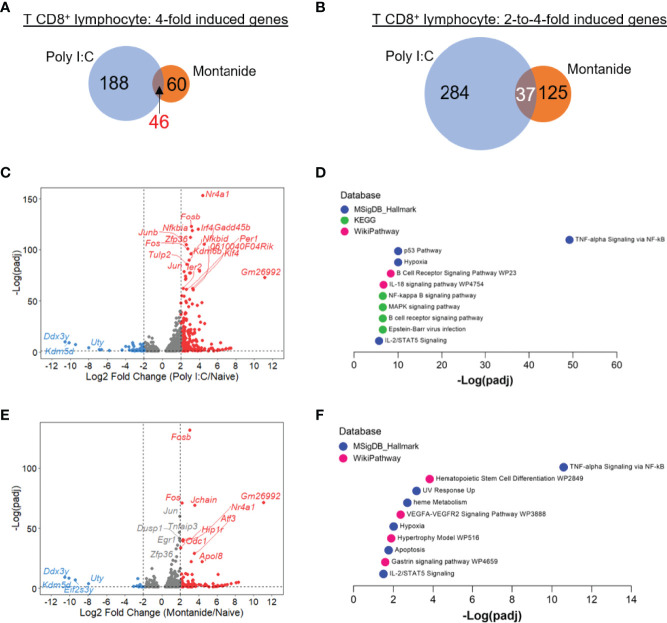
Poly (I:C) induced stronger perturbation in T CD8^+^ lymphocyte transcriptome in comparison to Montanide ISA 720. Differentially expressed genes (DEGs) were determined with the R-Bioconductor DESeq2 package, using the raw reads from the different samples as input. Genes that displayed a p-adjusted < 0.05 were considered for downstream analysis. **(A, B)**, Venn mulsionMontanide-adjuvanted or in both groups of mice. **(C)**, volcano plot highlighting the Poly (I:C)-induced DEGs. **(D)**, gene enrichment analysis of the 4-fold-induced genes by Poly (I:C), performed with the Enrichr webtool. **(E)**, volcano plot highlighting the Montanide-induced DEGs. **(F)**, gene enrichment analysis of the 4-fold-induced genes by Montanide ISA 720, performed with the Enrichr webtool. e.

Looking to CD8^+^ T lymphocytes transcriptome, we could detect a more pronounced difference in the effect of both adjuvants. Both the numbers of 4-fold induced, and 2-to-4-fold induced genes were higher in the Poly (I:C) sample in comparison to Montanide. Poly (I:C) induced the expression of 188 highly induced genes, while Montanide induced de expression of just 60 genes (46 genes were 4-fold induced by both adjuvants; **Figure 5A**). In the case of moderately induced genes, Poly (I:C) induced 284 while Montanide induced 125 genes (37 genes were moderately induced by both adjuvants, **Figure 5B**). These diffeneces are also showed in Volcano plots (**Figures 5C, E** for Poly (I:C) and Montanide respectively.

Regarding Gene Enrichment, “TNF-alpha signaling *via* NF-κB” was again the most prominent category for both adjuvants, and again the significance was much higher for Poly (I:C) (**Figures 5D, F**). The terms “Hypoxia”, “B cell receptor signaling pathway” and “IL-18 signaling pathway” were among the most significant ones in the Poly (I:C) sample, while “Heme metabolism” appeared again in the Montanide sample. Like it happened for B cells, GSEA analysis generally led to same conclusions of Enrichr analyzes for both T cell populations (**Supplementary Figures 2C–F**). We also performed HOMER motif analyzes for the 3-fold induced genes for both T cell populations and for both adjuvants, but the retrieved results were poorly significant and/or did not cross the threshold of false discovery rate (p adjusted < 0.05, data not shown).

### Antigen Presence Had Marginal Effects on the Transcriptome of Lymphocyte Populations

We then asked if the perturbations in the transcriptome of the different lymphocyte populations could be somehow modulated by the presence of the PvCSP protein. For each lymphocyte population and each adjuvant, we sorted the expressed genes in three groups: 1) genes that were significantly regulated by any adjuvant in comparison to naïve sample (defined by a p-adjusted < 0.05 according to DESeq2 analysis); 2) genes that were regulated by the adjuvant and further regulated by the presence of the antigen (that is, genes that showed a p-adj < 0.05 for the fold change where the numerator is the sample from mice immunized with Adjuvant+Protein and the denominator is the sample derived from mice injected with Adjuvant only); and 3) genes that were not regulated by the adjuvant, but showed statistically significant modulation in the sample from mice immunized with Adjuvant+Protein in comparison to Adjuvant only.

The results from these analyzes are depicted in **Supplementary Figure 3**. For items A to C, the left panel is always the set of genes modulated by the adjuvant but not modulated by the protein (group 1); the middle panel is the set of genes modulated by the adjuvant AND modulated by the protein (group 2); and the right panel is the set of genes that were not modulated by the adjuvant but were modulated in the sample where the protein was present (group 3). As we can observe, irrespectively of the adjuvant, cell population or if the gene is modulated according to the DESeq2 analysis, the level of fold induction considering the naïve sample as the baseline is essentially the same regardless the presence of the protein. Taken together, these results show that the main factor that modulates the transcriptome of the lymphocyte populations is the adjuvant.

## Discussion

In the present study, we further explored the differences in the immune response induced by vaccine formulations targeted to the Circumsporozoite protein from *P. vivax.* These formulations included different adjuvants, namely the TLR-3 agonist Poly (I:C) and Montanide ISA 720. Our previous articles have shown the efficacy of formulations that included both adjuvants, with a greater protective capacity observed when Montanide was present in the formulation (6–9). The results presented here not only reinforce the previous findings since Montanide formulations could induce higher titers of PvCSP-specific antibodies, but they also suggested a better antibody response since antibodies generated in Montanide-adjuvanted mice showed increased avidity (**Figure 1**).

The implementation of vaccine formulations containing recombinant proteins instead of whole inactivated organisms, known as subunit vaccines, offers great advantages, including lower risk of side effects and ease manufacturing. However, an important disadvantage is that these recombinant proteins frequently present poor immunogenicity and fail to induce strong and long-lasting immune responses. Adjuvants have been traditionally used to increase the magnitude of an adaptive response to a vaccine, based on antibody titer or ability to prevent infection. A second role for adjuvants has become increasingly important: guiding the type of adaptive response to produce the most effective forms of immunity for each specific pathogen (34).

The class of adjuvant used in vaccine formulations generates different pattern of response, leading to outcomes that facilitate the prevalence of cell-mediated immunity or antibody responses (34). Particularly, broad and balanced responses would be optimal for a vaccine against malaria, although it is known that strong antibody responses against specific antigens play a crucial role. For this reason, Poly (I:C) is an attractive adjuvant choice for malaria vaccine approaches, as it can induce high and long-lasting antibody titers against *P. vivax* antigens (7), as well as partial protection in immunized mice (6, 8, 9, 14).

In studies following RTS,S/AS01E vaccination outcomes, it was hypothesized that antibody subclass and avidity measurements combined with specificity would associate with protection status. Protective immunity was mostly associated with the cytophilic subclasses (IgG1 and IgG3 in humans, IgG2 in mice) and multiple repeating sequences present in RTS,S formulation were considered to enhance the avidity of the antibodies (35). Also, a correlation of protective immunity was suggested to more prolonged antigen exposure, facilitating response to pre-erythrocytic antigens (36).

Oil-based emulsions adjuvants elicit high-avidity antibodies through benefit affinity maturation and differentiation upon antigen recall (37). In agreement, Montanide triggered higher cytophilic-type IgG titers (IgG2b) and was able to generate antibodies with higher avidity than those elicited by Poly (I:C). These differences could explain the higher partial protection against the challenge that Montanide conferred when administered with a PvCSP-based antigen (10). However, the protection achieved was not sterile. This could be related to the even higher IgG1 titers that both adjuvants elicit; since passive transfer experiments showed that IgG1 antibodies failed to protect BALB/c mice from challenge with malaria parasites. A possible explanation of the failure in protection is the preferential binding of murine IgG1 to inhibitory FccRIIB (CD32B), absent in neutrophils (38). For these reasons, our results lead us to suggest that adjuvants that trigger a response that minimizes the production of murine IgG1 and exacerbates IgG2 or IgG3, will result in formulations with greater success in preventing the disease, even using antigens that have already shown promise in invasion inhibition assays but that have failed to protection in animal models or clinical trials.

The apparent success of Montanide ISA 720 in evoking a protective immune response at least in the murine model against malaria infection, is lessened by the reactogenic effects of this oil-based adjuvant. Even though such reactogenicity could hamper its further use in clinical trials, it is important to analyze the immunological profile that triggered some degree of protection. In this way, knowledge about adjuvant activated pathways could help choose a new formulation that would elicit a similar immune profile without the adverse effects observed.

Analyzing the cytokine profile, Montanide-adjuvanted mice showed increased circulating levels of TNF-α and IL-9 (**Figure 2**). IL-9 was initially described as a Th2 cytokine since its production correlated with the expansion of antigen-specific Th2 cells (39, 40). Later, it was observed that cells specialized in IL-9 production that did not secrete the other hallmark Th2 cytokines (IL-4, IL-5, and IL-13) could be differentiated *in vitro* in the presence of IL-4 and TGF-β (41, 42). These cells were named Th9 cells and, although also detected *in vivo*, there is still a controversy in whether Th9 cells represent a stable T helper subpopulation or if it is a transient population that appears in the course of immune responses due to T cell plasticity (43). Other cell types have been described as sources of IL-9, like mast cells (44), group 2 innate lymphoid cells (ILC2s) (45), T follicular helper cells (46), Tregs (47), and memory B cells (48).

IL-9 also plays a significant role in allergic processes. For example, mast cells represent an important cellular target for IL-9 since this cytokine stimulates mast cell proliferation and the secretion of other inflammatory mediators, like IL-6 (49, 50). In patients with asthma, mast cells were the major population of cells that expressed IL-9 receptors (51). These reports from the literature could lead us to speculate that the great reactogenicity observed in Montanide-adjuvanted individuals could be linked to the increased circulating levels of IL-9.

On the other hand, some effects of IL-9 could be helpful in vaccination models, since important roles for this cytokine were described in the development of memory B cells. Wang and colleagues have shown that IL-9 derived from follicular helper T cells (T_FH_) is essential for the differentiation o memory B cells since the specific deletion of IL-9 production by T_FH_ impairs that differentiation (46). Later, Taktsuka and colleagues showed that mice deficient in IL-9R had shown normal primary antibody response but reduced recall response and reduced differentiation of Plasma Cells from memory B cells (48). We do not have experimental evidence that links the increased systemic levels of IL-9 to the eventual local production of this cytokine in germinative centers. Nevertheless, the significance of this systemic production of IL-9 is certainly worth investigating in the future to understand better its role in vaccination models.

Interestingly, Poly (I:C) also favored a balanced cytokine profile, with some characteristics of an inflammatory allergic-type response. As mentioned, this adjuvant induced higher levels of IL-13, a pleiotropic type 2 cytokine that has been shown to be integral in the pathogenesis of asthma and other eosinophilic disorders (52). Poly (I:C)-adjuvanted groups also showed a tendency to produce Eotaxin, a chemokine that mediates eosinophil recruitment in allergic inflammation (53). Under our experimental conditions we could not detect the production of inflammatory cytokines in Poly (I:C)-adjuvanted samples. Nevertheless, the transcriptomic analysis showed that Poly (I:C) induced an enrichment of genes related to inflammatory responses. Among them, the pathways TNF-alpha signaling *via* NF-κB and Hypoxia were significantly up-regulated in both B- and CD4^+^ T-lymphocytes. The pathways that regulate metabolic changes in activated lymphocytes usually depend on the activation of the HIF-1α transcription factor (54, 55). Moreover, HIF-1α may also be regulated in response to inflammatory stimuli in an NF-κB–dependent manner (56).

Another interesting finding relative to B cells that emerged from the transcriptome analysis is the “heme biosynthesis” gene signature prominent in the Montanide sample. This fact may indicate that the differentiation of Plasma Cells is favored in Montanide-adjuvanted mice. This point deserves special attention since it was demonstrated that RTS,S vaccine-elicited CSP-specific antibody titers are associated with protection and with enhanced expression of genes related to differentiation of Plasma Cells (35).

B cells from Montanide-adjuvanted mice showed increased expression of the transferrin receptor TFRC-1, which mediates the immune cells uptake of transferrin-bound iron from serum (27). Iron uptake, in turn, is closely related to the activation of Heme biosynthesis (57). It has been shown that Heme physically interacts with Bach2, an essential transcription factor that regulates the fate of B lymphocytes. Bach2 represses the expression of the *Prdm1* gene encoding the Blimp-1 transcription factor, which expression triggers the differentiation of Plasma Cells. It has been proposed that Bach2 fine-tunes the differentiation of Plasma Cells, delaying this differentiation to allow the B lymphocytes to perform antibody class switch and affinity maturation. The addition of exogenous Heme to B lymphocyte cultures accelerates the activation of Blimp-1, therefore accelerating the differentiation of Plasma Cells that tend to secrete IgM (26).

More recently, Tsui and collaborators have unraveled the mechanisms through which PKCβ regulates B cell fate. They observed that PKCβ is essential for forming germinative centers (GCs) and Plasma Cell differentiation. Mice deficient in this kinase have defects in antigen polarization and defective activation mTORC1, a mediator of the metabolic reprogramming and mitochondrial remodeling that ultimately leads to heme biosynthesis (31). Also, Price and collaborators evaluated the epigenetic landscape of memory B cells in mice and humans. They found that genes involved in Plasma Cell differentiation showed accessible chromatin in their surroundings. The heme biosynthesis signature is also conserved between rodents and humans, and treatment with Hemin promoted the changes in the metabolism of memory B cells, stimulating oxidative phosphorylation and differentiation to Plasma cells (57).

Even though Poly (I:C) triggered more perturbations in T cell transcriptome, the phenotype of the populations induced by both adjuvants showed some similarities. For example, the pathway TNF-alpha signaling by NF-κB was the most prominent term for both CD4^+^ and CD8^+^ T cells, suggesting that inflammatory responses after immunizations with both adjuvants could be mediated by the same mechanisms in these cells. Usually, TNF induces activation of multiple signaling pathways, including NF-κB and MAPK, promoting the induction of inflammatory gene expression (58). Moreover, CD4^+^ T cells from both Montanide and Poly (I:C)-adjuvanted samples showed enrichment of TGF-β and IL-18 pathways. Even though IL-18 is considered an immunostimulatory cytokine, it presents an additive effect on regulatory functions of TGF-β1 in cancer model, affecting the cytolytic activity of NK cells (59). Therefore, these pathways could act as regulators of the immune response elicited by the adjuvants in T cells.

Collectively, our results contribute to increasing the understanding of Poly (I:C) and Montanide ISA 720 mechanisms of action. Montanide may still have a potential for clinical use in vaccine formulations against malaria, since it can contribute to the generation of high avidity antibodies and seems to favor the differentiation of antibody-secreting Plasma cells. Our results suggest that, even though incorporating other immunomodulatory molecules could improve the outcomes and favor an approach that activates both the cellular and humoral components, the signaling pathways activated by adjuvants would define the immune profile that a formulation will elicit. Also, it would be important to unravel the mechanisms of action of vaccine formulations that confers sterile protective immunity. Improving our understanding of those mechanisms will provide insights into how to develop better vaccines against malaria and other infectious diseases.

## Data Availability Statement

The datasets presented in this study can be found in online repositories. The names of the repository/repositories and accession number(s) can be found below:https://www.ncbi.nlm.nih.gov/geo/, accession ID: GSE203218.

## Ethics Statement

The animal study was reviewed and approved by CEUA/FCF 74.2016-P531.

## Author Contributions

DB, IS, and AG contributed to conception and design of the study. RM, FM, JN and AG performed the experiments, data collection and analysis. RM, FM, and AG wrote the manuscript. All authors contributed to manuscript revision, read, and approved the submitted version. All authors contributed to the article and approved the submitted version.

## Funding

RM, JN, IS and AG were supported by Fundação de Amparo à Pesquisa do Estado de São Paulo (FAPESP) fellowships (RM, #2021/04455-9; JN, #2018/25993-6; AG, #2014/18102-7) and grant (IS, #2012/13032-5). IS received fellowships from CNPq. FM, DB and AG were supported by Serrapilheira (Grant #G-1709-16618).

## Conflict of Interest

The authors declare that the research was conducted in the absence of any commercial or financial relationships that could be construed as a potential conflict of interest.

## Publisher’s Note

All claims expressed in this article are solely those of the authors and do not necessarily represent those of their affiliated organizations, or those of the publisher, the editors and the reviewers. Any product that may be evaluated in this article, or claim that may be made by its manufacturer, is not guaranteed or endorsed by the publisher.
