# Supplementary material for: Immune System Modulation by the Adjuvants Poly (I:C) and Montanide ISA 720
Source: Front Immunol. 2022 Jun 29;13:910022. doi: 10.3389/fimmu.2022.910022 (PMC9278660; doi:10.3389/fimmu.2022.910022)
Supplement: Supplementary file 1 [file DataSheet_1.pdf]

Supplementary Figure 1

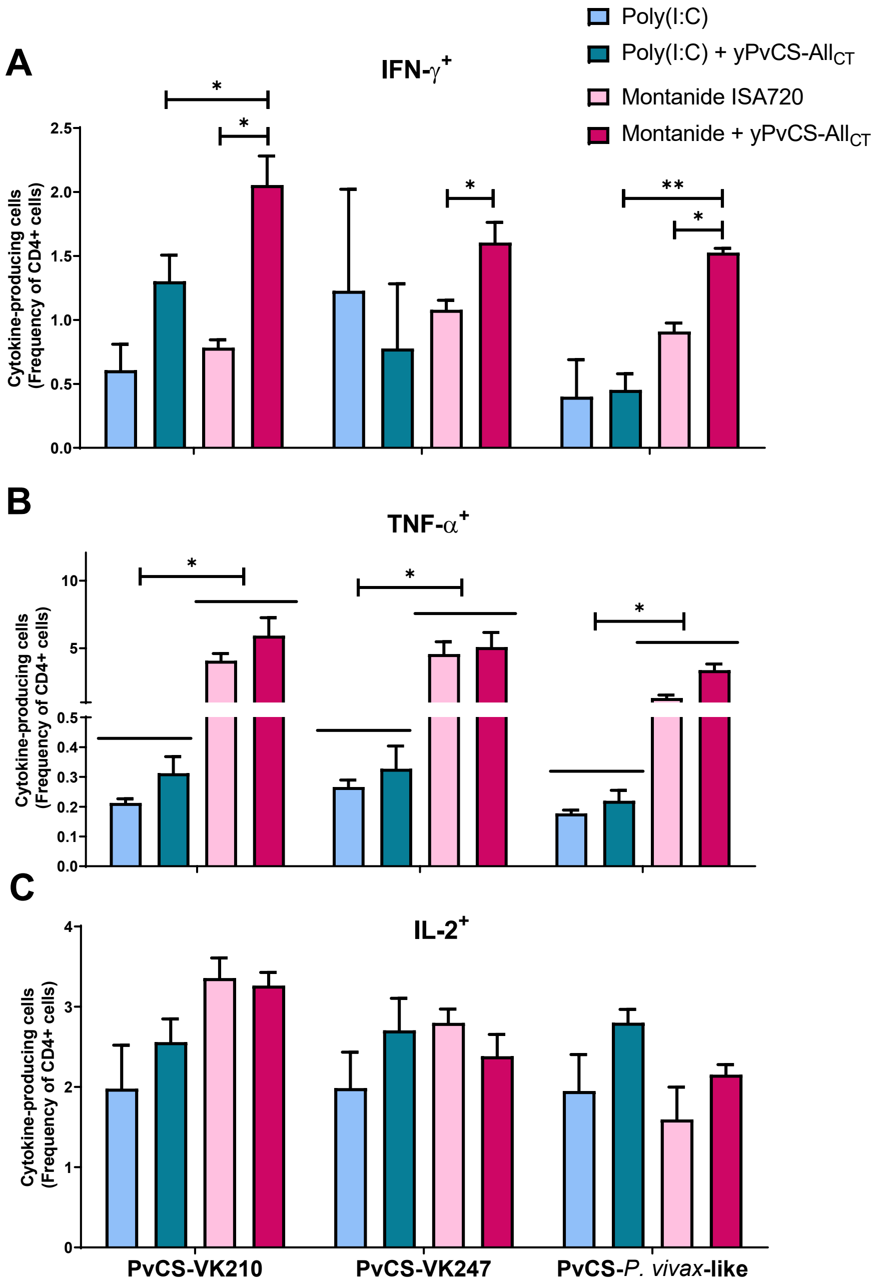

Supplementary Figure 1 – Montanide induces PvCSP-specific T CD4^{+} lymphocyte clones that produce inflammatory cytokines. Splenocytes from pooled mice (n = 6 animals/ group) were stimulated ex vivo with 10 µg/mL of the recombinant proteins PvCS-VK210, PvCS-VK247 or PvCS-*P. vivax*-like and incubated in the presence of brefeldin for 12–16 h. Graphs show the percentage of cells producing IFN-γ, IL-2, or TNFα in the CD3^{+}CD4^{+} gate after subtraction of values obtained in the absence of any stimulus. Bars indicate mean ± SEM. One representative experiment of two is depicted.
