# Supplementary material for: Immune System Modulation by the Adjuvants Poly (I:C) and Montanide ISA 720
Source: Front Immunol. 2022 Jun 29;13:910022. doi: 10.3389/fimmu.2022.910022 (PMC9278660; doi:10.3389/fimmu.2022.910022)
Supplement: Supplementary file 2 [file DataSheet_2.pdf]

Supplementary Figure 2

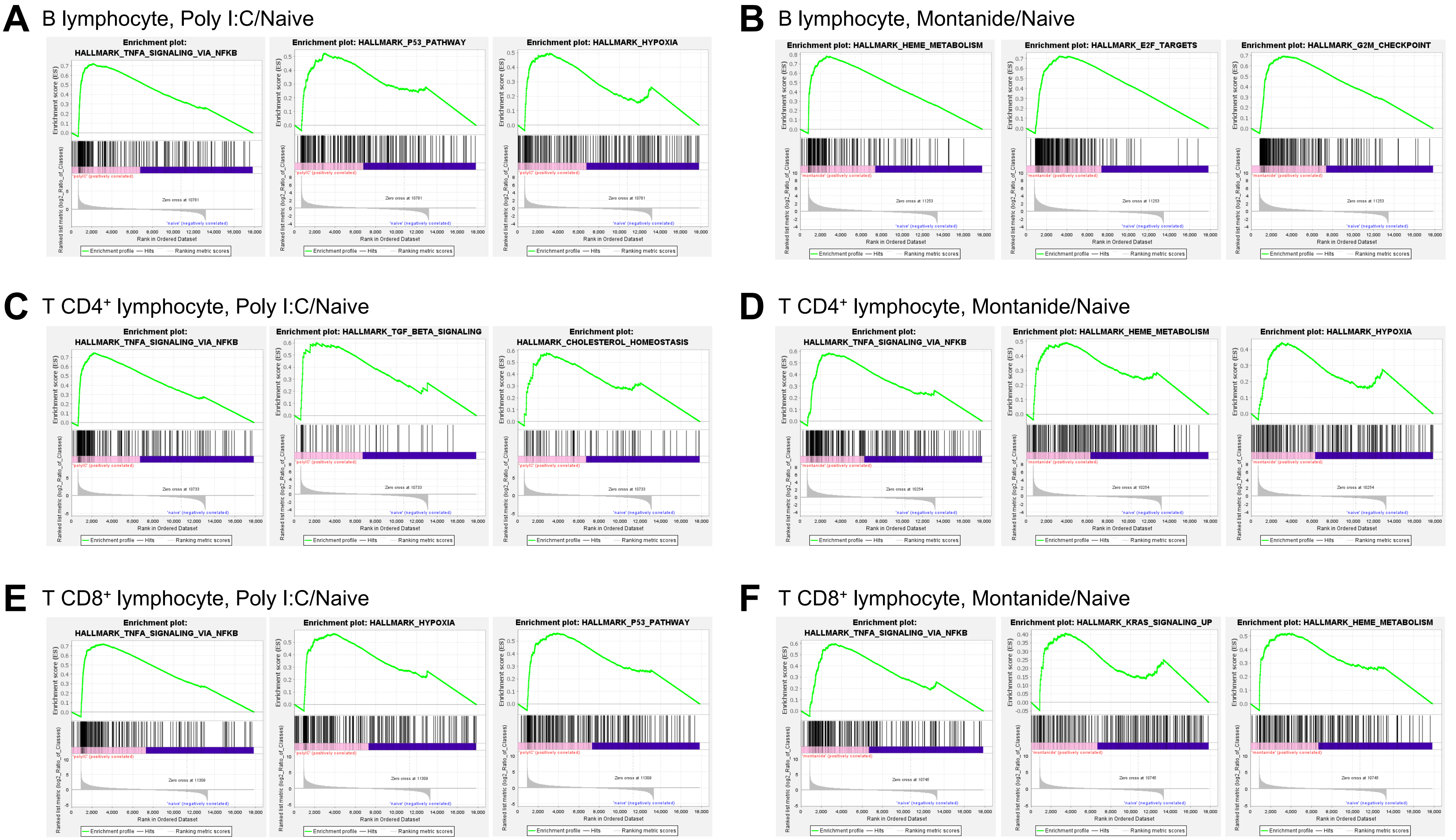

Supplementary Figure 2 – GSEA analysis of lymphocyte populations transcriptomes. Normalized reads from all samples were obtained with R-Bioconductor DESeq2 package. The lists of these reads were submitted to the GSEA software (Broad Institute/UCSD) and the Enrichment Score (ES) plots from the three top terms in each sample were included here.
