# Supplementary material for: Immune System Modulation by the Adjuvants Poly (I:C) and Montanide ISA 720
Source: Front Immunol. 2022 Jun 29;13:910022. doi: 10.3389/fimmu.2022.910022 (PMC9278660; doi:10.3389/fimmu.2022.910022)
Supplement: Supplementary file 3 [file DataSheet_3.pdf]

Supplementary Figure 3

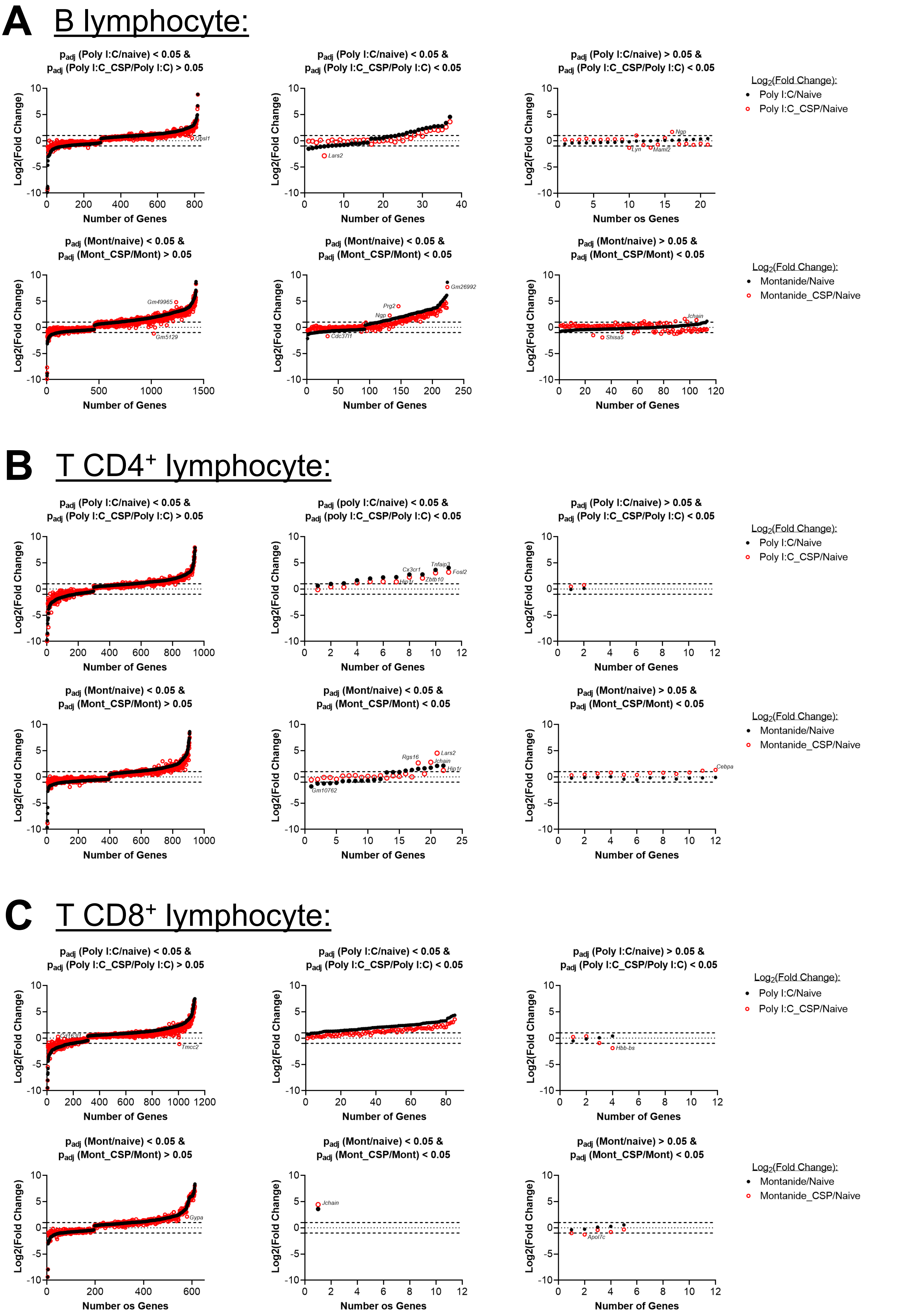

Supplementary Figure 3 – Presence of antigenic protein marginally influence the adjuvant effects on DEGs. Differentially expressed genes (adjuvant *versus* naïve, adjuvant+antigen *versus* adjuvant and adjuvant+antigen *versus* naïve) were obtained with R-Bioconductor DESeq2 package. Genes from each tested condition were split into three major groups: 1) genes significantly induced by adjuvant (over naïve) and not significantly induced by adjuvant+antigen (over adjuvant); 2) genes significantly induced by adjuvant (over naïve) and significantly induced by adjuvant+antigen (over adjuvant); 3) genes not significantly induced by adjuvant (over naïve) and significantly induced by adjuvant+antigen (over adjuvant). The lists of DEGs from both "adjuvant *versus* naïve" and "adjuvant+antigen *versus* naïve" in the three major groups above mentioned, for each tested condition, were ranked from larger to smallest Log2(Fold Change) and these values were plotted as scatter plots. A gene was considered significantly regulated if p-adjusted < 0.05.
